# FTO effects the proliferation, invasion, and glycolytic metabolism of colon cancer by regulating PKM2

**DOI:** 10.1007/s00432-024-06073-x

**Published:** 2025-01-16

**Authors:** Kongyan Zhang, Fei Zhang, Jiahe Wang

**Affiliations:** 1 Department of Geriatrics, Fuyang Hospital of Anhui Medical University, Fuyang, 236000 Anhui China; 2 Department of Family Medicine, Shengjing Hospital of China Medical University, Shenyang, 110004 Liaoning China

**Keywords:** FTO, PKM2, Colon cancer, Glycolytic metabolism, Therapeutic target

## Abstract

**Purpose:**

Colorectal cancer (CRC) is a leading cause of cancer-related mortality worldwide. The Fat mass and obesity-associated protein (FTO), a genetic variant associated with obesity, significantly impact the energetic metabolism of mechanical tumors. However, research on the function of FTO in CRC is scarce.

**Methods:**

Bioinformatics analysis of TCGA and UALCAN databases was conducted to examine FTO expression in CRC. Immunohistochemistry was used to assess FTO and PKM2 protein expression in clinical specimens. In vitro experiments utilized five human colon cancer cell lines and a normal colon epithelial cell line, with Western blotting and RT-PCR for protein and mRNA quantification, respectively, and lentiviral transfection to modulate FTO expression. Cellular behaviors such as proliferation, migration, invasion, and apoptosis were evaluated using various assays. Immunofluorescence and Seahorse Xfe96 metabolic analysis were employed to study PKM2 expression changes and glycolytic stress. The effects of PKM2 inhibition by shikonin on cell viability and glycolytic activity were assessed using CCK-8 assay and Seahorse analysis.

**Results:**

An upregulation of FTO was observed in colon cancer through data mining and analysis of pathological specimens. Besides, we discovered that the impact of FTO on colon cancer glycolysis has significant implications for colon proliferation, invasion, and metastasis. The protein expression of PKM2 and the intensity of fluorescence staining in the nucleus of PKM2 were detected to be increased in colon carcinoma cells with over-expression of FTO.

**Conclusion:**

FTO plays a significant role in CRC progression by regulating PKM2 and promoting glycolysis.

**Supplementary Information:**

The online version contains supplementary material available at 10.1007/s00432-024-06073-x.

## Introduction

Colorectal cancer (CRC) ranks as the second most prevalent cause of tumor-related mortality worldwide, while its global incidence stands as the third highest among all tumors(Ke et al. 2024). Notably, the incidence of CRC in individuals under 55 is steadily rising at an annual rate of 2%(Siegel et al. 2020; Sung et al. 2021). It is widely acknowledged that obesity engenders significant alterations in detrimental hormonal profiles(Chen et al. 2022) and metabolic immunity(Yuan et al. 2023) within the body, thereby augmenting the susceptibility to CRC. In conjunction with advancements in early screening, diagnosis, and diverse treatment modalities, the extended survival duration of colon cancer has been observed. However, the survival rate for individuals in high-risk stages has not experienced significant improvement. Consequently, it becomes imperative and time-sensitive to investigate the etiology of colon cancer further and identify novel biomarkers and therapeutic targets.

FTO, an allele linked to obesity, is situated on the elongated segment of human chromosome 16, specifically 16q12.2. Its gene length is approximately 400 Kb, encompassing nine exons and eight introns(Suthon and Tangjittipokin 2024). FTO has emerged as a potential biomarker in cancer research due to its critical role in RNA epigenetics, particularly in regulating m6A demethylation(Li et al. 2024). Its dysregulation has been linked to various cancers, making it a promising candidate as a tumor biomarker. Li et al. conducted a study on AML and found that up-regulation of FTO can contribute to tumor progression by influencing the overall level of m6A(Li et al. 2017). However, the investigation of FTO in colon cancer remains limited.

The distinctive feature of colon tumor cells is their abnormal proliferation, necessitating energy metabolism modulation to meet their unrestricted growth demands. Consequently, exploring energy metabolism regulation has become a prominent area of research in understanding the mechanisms underlying tumor development. PKM2, an isoform of PKM(Khedr et al. 2024), predominantly expressed in embryonic cells, stem cells, and tumor cells characterized by heightened anabolic requirements, is a crucial enzyme in the metabolic process of aerobic glycolysis in neoplastic growths. The primary objective of this study was to examine the potential influence of PKM2, a crucial molecule downstream of PFKP in the glycolytic pathway, on the regulation of PKM2 expression via the involvement of FTO in PFKP. This investigation sought to determine whether such an interaction could enhance the rate of aerobic glycolysis in tumors. To address this knowledge gap, the study aimed to investigate how FTO affects the malignant biological behavior and aerobic glycolytic metabolism of colon cancer by regulating the expression of PKM2, focusing on the perspective of energy metabolism.

## Materials and methods

### TCGA database

The RNA-seq data about colon carcinoma were obtained from the data portal of The Cancer Genome Atlas (TCGA) at https://tcga-data.nci.nih.gov. Afterwards, the genetic information was transformed into transcripts per million reads (TPM) format and underwent a logarithmic conversion. The data analysis was performed utilizing VisualSonics software, specifically R Studio (Version 3.6.3). The generation of graphs was accomplished by utilizing R packages ggplot2 and GSVA.

### UALCAN database

The UALCAN database, accessible at http://ualcan.path.uab.edu, analyses cancer omics data using TCGA level 3 RNA-seq and TCGA gene expression data from 31 cancer types. This database was developed to validate target genes and identify potential biomarkers for specific tumor subsets. In our research, we investigated the expression of FTO with different clinical parameters.

### Clinical specimens

103 clinical samples were acquired from the Department of Pathology at Shengjing Hospital of China Medical University between 2017 and 2019. These samples comprised 57 males and 46 females and were selected based on well-defined pathological diagnoses. All patients provided informed consent, and the local ethics committee approved the study by the Declaration of Helsinki (ethics approval number: 2022PS700K). Patient demographic and clinical data were collected using an electronic case system. The colon tumors included in the study were untreated before surgery and were all classified as primary tumors. Following the guidelines of the 8th edition of the AJCC system, patients were staged for TNM staging.

### Immunohistochemistry

Immunohistochemistry was conducted on paraffin-embedded tissues fixed in a 4% paraformaldehyde solution and subsequently sliced into 3 μm sections using a conventional microtome. The sections were subjected to dewaxing with xylene, followed by hydration with gradient ethyl alcohol. Subsequently, the sections underwent antigen heat retrieval in a microwave and were allowed to cool naturally. After being incubated in 10% goat serum, the sections were subjected to overnight incubation at 4 °C with the monoclonal rabbit anti-human FTO antibody (ab126605, Abcam) at a dilution of 1:50. Subsequently, the sections were exposed to a goat anti-rabbit secondary antibody at 37 °C for 25 min. Following three washes with PBS, color development was achieved using diaminobenzidine (DAB). Subsequently, following counterstaining with hematoxylin, the nuclei exhibited a blue hue. Subsequently, the sections were dehydrated using alcohol and xylene gradients, then dried and sealed with neutral gum. As an alternative to antibodies, phosphate-buffered saline was employed as a negative control.

### Cell lines

In this study, a series of in vitro experiments were performed using five distinct cell lines of human colon cancer, namely LOVO, SW620, SW480, Caco-2, and CL187, along with a human standard colon epithelial cell line known as HcoEpic. The LOVO, SW620, SW480, and Caco-2 cell lines were obtained from the Cell Resource Center in Shanghai, China, affiliated with the Shanghai Institute of the Chinese Academy of Sciences. The CL187 cell line, on the other hand, was obtained from ScienCell. Moreover, the HcoEpic cell line was purchased from the American-type culture collection.

### Western blotting

The obtained cellular proteins were analyzed for protein quantification using the BCA technique. Subsequently, the proteins were denatured, subjected to electrophoresis in pre-configured gels, and transferred onto a PVDF membrane. Then, it was sealed with 5% skim milk powder for 3 h. Following this, the FTO primary antibody (ab126605, Abcam) was applied at a concentration of 1:10,000 and left to incubate overnight at a temperature of 4℃. After being rewarmed to room temperature, the membrane underwent three washes with TBST. Subsequently, the secondary antibody (1:5000) was administered and left to incubate at room temperature for 1.5 h. Ultimately, the luminescent solution was employed to facilitate development, and the outcomes were assessed with software assistance.

### Reverse transcription-polymerase chain reaction

The cellular RNA was quantified after extraction, followed by the generation of cDNA using reverse transcriptase. The cDNA obtained was then utilized in RT-PCR experiments using the Applied Biosystems 7500 Real-Time PCR System method. The internal reference PGK1 was employed for normalization. The primer sequences for FTO were forward, 5'-GCCGCTGCTTGTGAGACCTTC-3', and reverse, 5'-TGCTGCTCTGCTCTCTTAATGTCCAC-3'. Similarly, the primer sequences for the internal reference PGK1 were as follows: forward, 5'-TTCTGTTCTTGAAGGACTGTGT-3', and reverse, 5'-CTTTAACCTTGTTCCCAGAAGC-3'.

### Lentiviral transfection

The lentivirus encoding FTO knockdown, FTO overexpression, and empty vector lentivirus were synthesized by Shanghai Heyuan Company. Lentiviral transfection was conducted according to the manufacturer's instructions. The target RNAi sequences used were as follows: shFTO#1, GGAGCTCCATAAAGAGGTT; shFTO#2, GCAGCATACAACGTAACTT; shFTO#3, GGTGGCAGTGTACAGTTAT. Colon cancer cells exhibiting high FTO expression were subjected to FTO knockdown, whereas colon cancer cells displaying low FTO expression underwent FTO overexpression. Subsequently, stably transfected cells were screened and cultivated with 2 ug/ml puromycin (Sigma-Aldrich, USA) for 72 h.

### CCK8 assay

The cell growth ability was evaluated by performing the CCK-8 assay. The experimental cells were placed in 96-well plates with 3 replicate wells per sample and incubated in a cell culture incubator at 37 ℃ with 5% CO2 for durations of 0 h, 24 h, 48 h, 72 h, and 96 h. Subsequently, the samples were exposed to CCK-8 for 2 h, and the optical density (OD) reading at 450 nm was measured using zymography and recorded.

### Scratch assay

A scratch assay was performed to evaluate the migratory characteristics of colon cancer cells. A 2 ml cell suspension from each experimental group, containing approximately 50 × 10^5^ cells per well, was inoculated into every well of a 6-well plate. Subsequently, the 6-well plate was incubated overnight in a cell culture incubator at a temperature of 37℃ with a 5% CO2 environment. The following day, the 6-well plate was retrieved from the incubator, and the cells were examined using an inverted microscope to assess their distribution within each well. Upon confirmation of cell spreading, a 100 µl tip was used to draw a straight line on the cell monolayer. Subsequently, the serum-free medium was replaced, and the plate was placed in a 37℃ cell incubator containing 5% CO2 for cultivation periods of 0 h, 24 h, and 48 h. Finally, the cells were observed under a microscope. Cell migration capacity was assessed by calculating the healing rate of traumatic wounds, which was determined as the migratory distance divided by the original surface distance multiplied by 100%.

### Cell invasion assay

Cell invasion experiments were conducted utilizing a Matrigel® encapsulated invasion chamber (BD Biosciences). A 200 µl of cell suspension from each experimental group (approximately 1.0 × 10^5^ cells/well) was introduced into the upper chamber, while the lower chamber was supplied with a medium containing 10% FBS. Following a 48-h incubation period, the cells in the upper chamber were softly cleaned and treated with a 4% paraformaldehyde solution for 15 min at ambient temperature. Afterwards, these cells were stained with a 1% crystal violet solution and left at room temperature for 30 min. Upon completion of the staining process, the cells were rinsed with ddH2O 3–5 times to eliminate any residual crystal violet stain in the background. Additionally, any cells remaining on the chamber's edges were meticulously eliminated with a cotton swab. Finally, the cells were observed and documented under a microscope.

### Cell apoptosis assay

The Annexin V-APC/7-AAD Apoptosis Detection Kit was utilized to assess apoptosis in the various experimental groups of colon cancer. Cells from each experimental group were initially gathered, and the supernatant was removed after washing them with pre-cooled PBS. Subsequently, Annexin V-APC and 7-AAD were employed for dual staining. By the kit's instructions, 100 μl of 1 × buffer was introduced to each cell group to resuspend the cells. Subsequently, 5 μl of 7-AAD and 5 μl of Annexin V-APC were added and allowed to stain the cells for 15 min. Following this, 400 μl of 1 × buffer was added to achieve dilution, and the cells were gently mixed using a 1000 μl pipette gun. The resulting mixture was then transferred to the uptake tube of the flow cytometer and examined utilizing FlowJo software (version 7.6.1; FlowJo LLC).

### Immunofluorescence

The cells from each experimental group, containing a concentration of 5 × 10^4^ cells/ml, were placed onto 24-well plates with circular coverslips of 14 mm diameter. Subsequently, the plates were placed in an incubator for 24 h to facilitate further cultivation. Following this, the cells were washed thrice with PBS and then exposed to 4% paraformaldehyde in each well. The fixation process was carried out in a 37℃ incubator for 10 min. To enable permeabilization, 0.5 ml of 0.2% Triton X-100 was incorporated into each well and left for 10 min. Subsequently, the cells were enclosed using a 10% goat serum working solution for 30 min at ambient temperature. Following this, the primary antibody of PKM2 (AF5234, Affinity) was introduced to the coverslip at a dilution ratio of 1:100. The coverslip was then placed within a moist container and stored overnight in a cold room at a temperature of 4 °C to prevent exposure to light. The moist container was removed from the 4 °C cold room a subsequent day. After rinsing, the goat anti-rabbit fluorescent secondary antibody was carefully added drop by drop to the coverslip in a dilution ratio of 1:50. The coverslip was incubated at ambient temperature, shielded from light, for 1 h. Subsequently, the slide was treated with the anti-fluorescence quenching sealing solution, which included DAPI. A coverslip containing cells affixed to it was placed over the slide. The resulting specimen was then examined and documented using a fluorescence microscope.

### Seahorse Xfe96 metabolic analysis

A Seahorse XFe96 analyzer (Seahorse Bioscience, Billerica, MA) was used to perform metabolic analysis for glycolytic stress analysis. The day before the experiment, cells from each experimental group were inoculated onto Seahorse cell culture microtiter plates at a density of 1.0 × 10^4^ cells/80 ul per well. Subsequently, the cell culture microtiter plates were put into a cell culture incubator and cultured overnight at 37℃. The probe plate was hydrated by the operational instructions. Seahorse XF Base medium, enriched with l-glutamine (2 mM) and adjusted to a pH of 7.4, replaced the medium one hour before the experiment. The objective was to examine the impact of the subject substances on the fundamental activity and capacity of both glycolysis and the energy system within the mitochondria. A total of six replicates were measured, and the resulting data sets were subsequently analyzed using the XFe-96 software developed by Agilent.

### Cell viability assay

The CCK-8 assay was employed to assess the viability of colon cancer cells. The colon cancer cells with overexpressed FTO were cultured in 96-well plates. In contrast, blank wells (i.e., wells devoid of cells containing CCK-8 solution and medium) and control wells (i.e., wells containing cells containing CCK-8 solution and medium without treatment) were concurrently established. Additionally, three parallel replicate wells were arranged for each cell group. The cell suspension was aliquoted at 100 µl per well and then placed in a cell incubator for 24 h. Once the cells adhered to the culture vessel and achieved a confluence ranging from 70 to 80%, the initial medium was discarded and substituted with serum-free DMEM/F12 medium supplemented with varying concentrations (0.5, 1, 1.5, 2, 2.5, 5, 7.5 µM) of the PKM2 inhibitor, comfrey. Once the cells had adhered to the wall and achieved a confluence of 70% ~ 80%, the original medium was discarded and substituted with serum-free DMEM/F12 medium containing various concentrations (0.5, 1, 1.5, 2, 2.5, 5, 7.5 µM) of the PKM2 inhibitor comfreyatin. Afterwards, the cells were incubated for 24 h, 48 h, and 72 h. Subsequently, the cells were placed in a cell culture incubator for 2 h with the addition of 1/10th of the cell culture medium volume, CCK-8. The 450 nm OD was subsequently measured using an enzyme labeling apparatus.

### Statistical analysis

The experimental data was collected using mean ± standard deviation (x ± s) and analyzed by SPSS 25.0 software (Statistics Package for Social Science, SPSS 25.0). Independent samples t-test was used for between-group comparisons, whereas multifactorial analyses of the clinical data utilized a one-way chi-square test and logistic regression. The level of statistical significance was established at a P-value less than 0.05.

## Result

### The expression of FTO is increased in colon cancer

**Fig. 1:**
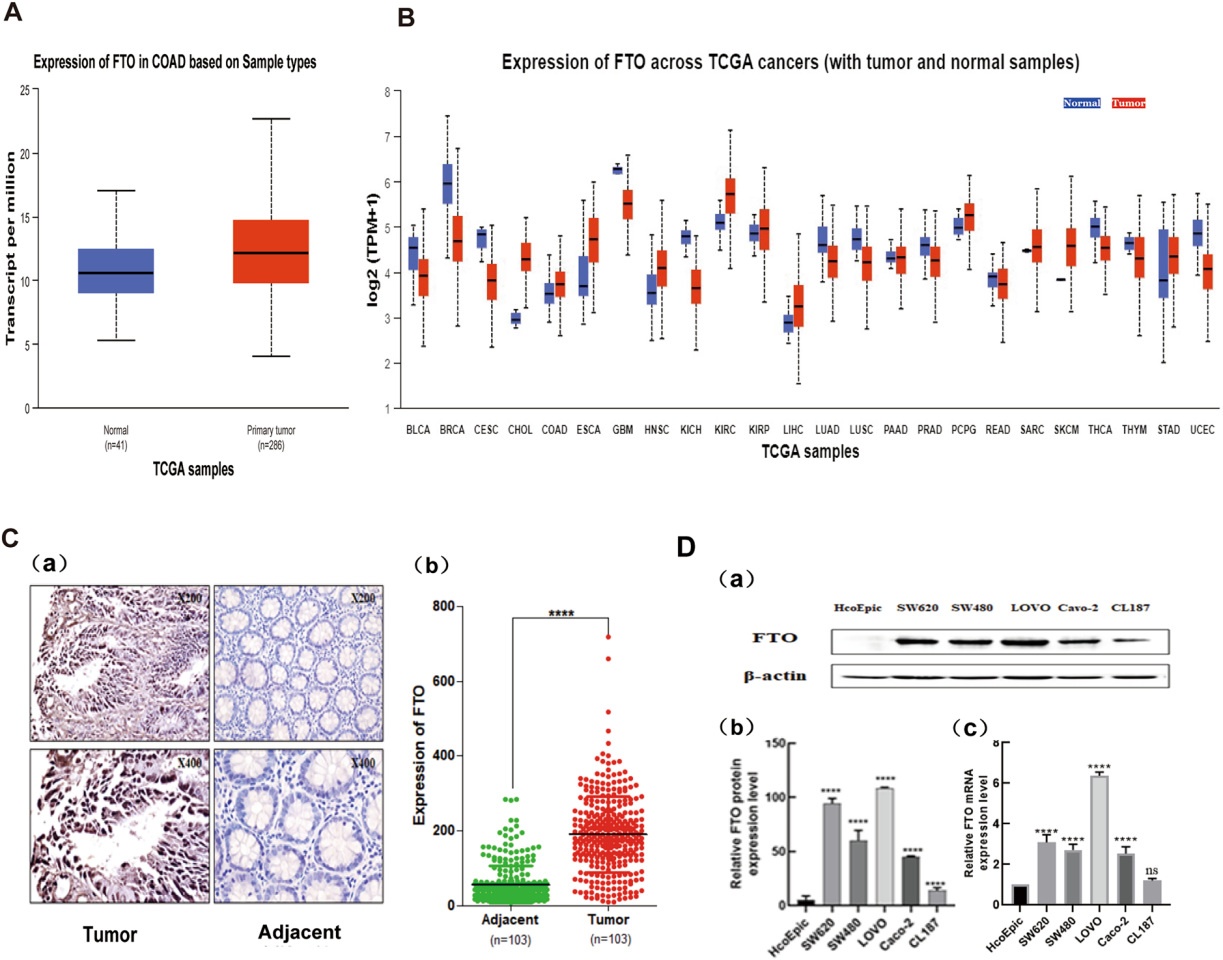
FTO is upregulated in colon cancer. **A**. FTO expression was higher in colon cancer tissues than in paracancerous tissues in TCGA database. **B**. FTO expression in various tumor tissues was significantly higher than that in adjacent tissues in TCGA database. **C**. High expression of FTO in human colon cancer tissues as confirmed by immunohistochemistry (a and b). **D**. In vitro experiments confirmed that five types of colon cancer cells , including SW620，SW480，LOVO，Caco-2，and CL187 cell lines，showed high expression of FTO at both the protein level and the RNA level compared to normal colon epithelial cells，HcoEpic cells (a,b ,and c).

The TCGA database was utilized to analyze the expression of FTO in pan-cancer and colon cancer and conduct a more comprehensive examination of FTO expression in tumours. The results revealed a significant elevation of FTO expression in various tumor tissues compared to adjacent tissues, a trend also observed in colon cancer. These differences were statistically significant, as depicted in Figs. 1A-B. Additionally, our study performed tissue validation using colorectal cancer pathology samples, as shown in Fig. 1C. FTO was predominantly localized within the nucleus, resulting in brown staining within cells exhibiting high expression levels. The findings of our study indicate that the levels of FTO expression in colon cancer tissues were notably elevated compared to adjacent tissues.

Furthermore, FTO expression was observed in colon cancer cells. The observation was confirmed by detecting FTO expression at protein and mRNA levels in five colon cancer cells and one normal colon epithelial cell. As illustrated in Fig. 1D, the expression of FTO was notably higher in the five different colon cancer cells at both the protein and mRNA levels compared with the normal colon epithelial cells. (Fig. 1D).

### The overexpression of FTO is correlated with the progression of clinicopathological characteristics in colon cancer

**Fig. 2:**
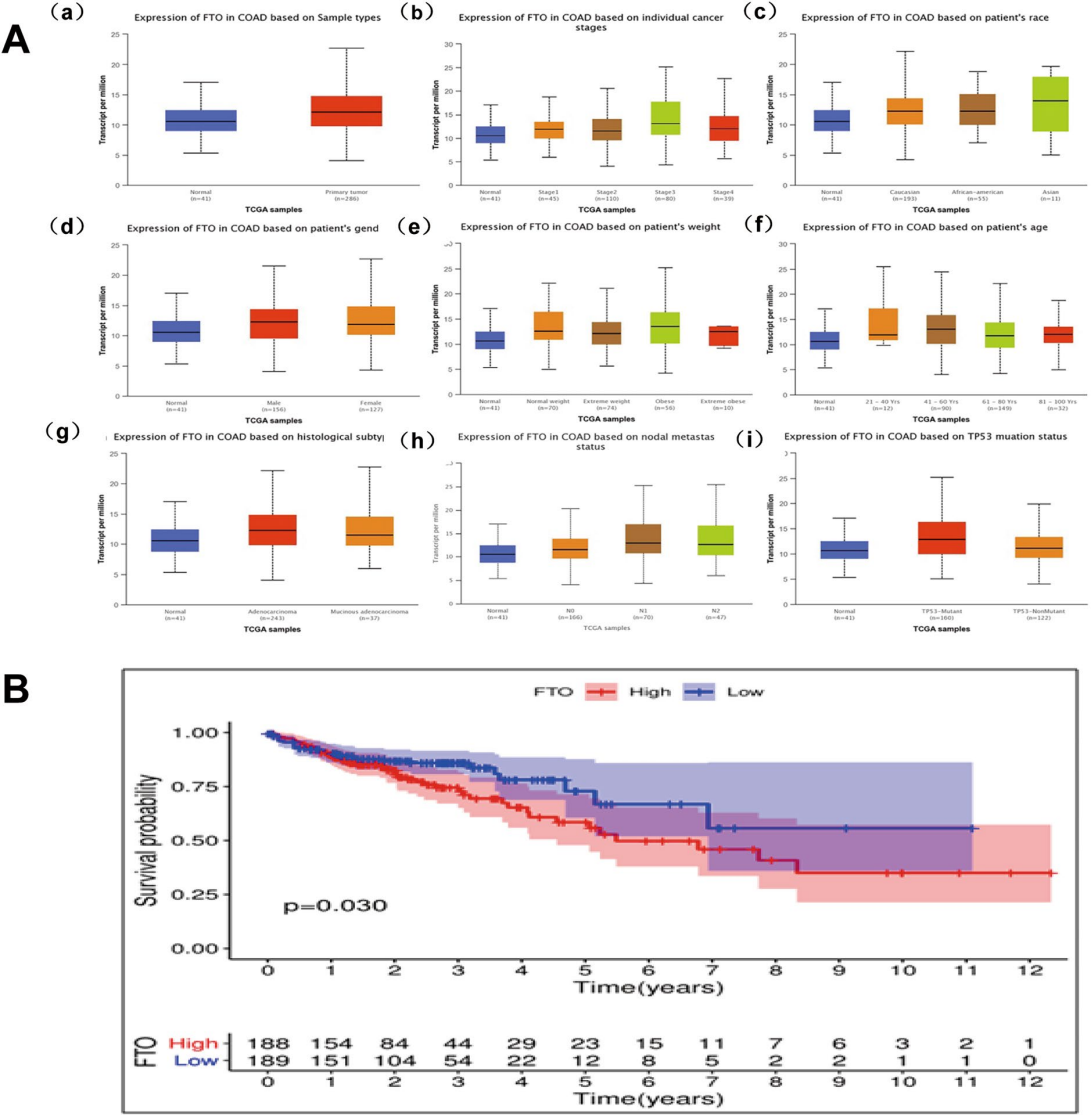
Upregulation of FTO is associated with advanced clinicopathological features of colon cancer. **A**. The relationship between FTO expression in colon cancer and clinicopathological features from TCGA and UALCAN databases. (a) sample types, (b) individual cancer stage, (c) patient race, (d) gender, (e) weight, (f) age, (g) histologic subtype, (h) nodal metastasis status, and (i) TP53 mutant status. **B**. Higher FTO expression was associated with a lower survival rate applying the TCGA database.

Our study investigates the correlation between FTO expression in colon cancer and clinicopathological characteristics using data from TCGA and UALCAN databases. Our findings indicate that several clinicopathological factors, such as sample types, individual cancer stage, patient race, gender, weight, age, histologic subtype, nodal metastasis status, and TP53 mutant status, have an impact on FTO expression in colon cancer (Fig. 2A). Moreover, the UALCAN database demonstrates a significant increase in FTO expression in colon cancer in comparison to normal tissue. The same result was confirmed in the analysis of 103 colon cancer tissues (Figs. 2A and 1C). A notable augmentation in the expression of FTO was observed in individuals diagnosed with clinical stage 3 of colon cancer, as compared to those diagnosed with clinical stage 2 and individuals without cancer (Fig. 2A). Among young women, FTO levels were significantly elevated in comparison to those of women without cancer (Fig. 2A). The degree of senescence exhibited by FTO was significantly greater in obese samples compared to the control group of individuals without obesity (Fig. 2A). A notable upregulation of FTO was observed in patients aged 21–40 and 41–60 years, compared to individuals with normal FTO expression levels (Fig. 2A).

Furthermore, histologic subtypes of colonic adenocarcinoma exhibited significantly elevated FTO expression compared to adjacent normal tissues (Fig. 2A). Patients with lymph node metastases N1 and N2 also displayed heightened FTO expression levels compared to those without metastases and the average population (Fig. 2A). An upregulation of FTO expression was observed in individuals harboring TP53 mutations, compared to those without TP53 mutations and those with normal TP53 status (Fig. 2A). Additionally, the prognostic evaluation conducted on colon cancer patients utilizing the TCGA database demonstrated that heightened FTO expression was correlated with a decreased survival rate (Fig. 2B).

Additionally, to validate the findings above within the database, an analysis was conducted to assess the expression of FTO within colon cancer tissues. This investigation involved the examination of FTO expression in a total of 103 colon cancer tissue samples through the utilization of immunohistochemistry. The study cohort consisted of 57 male and 46 female participants. Among these individuals, 30 (29.1%) exhibited the highest pathological grading (grades III and IV), while 73 (70.9%) displayed the lowest pathological grading (grades I and II) (Table 1). The analysis revealed that among the samples examined, 11 exhibited low FTO expression, while 92 exhibited high FTO expression (Fig. 1 C). These findings strongly suggest a significant association between FTO expression and various factors, such as age (P = 0.028), gender (P = 0.048), pathological T staging (P = 0.017), and tumor grade (P = 0.001). Nevertheless, no noteworthy correlations were found regarding the tumor size, clinical N status, clinical M status, vascular invasion, perineural invasion, serum CEA level, and CA-199 level (Table 2). Furthermore, the findings of multivariate Cox regression analysis demonstrated that tumor grade independently served as a risk factor for colon cancer (Supplementary Table 1).

**Table 1:** Clinical characteristics of patients in colon cancer

Characteristics	No.patients
Age(year)	
≥ 65	52(50.5)
< 65	51(49.5)
Gender	
Male	57(55.3)
Female	46(44.7)
Tumor size	
≥ 5cm	53(51.5)
< 5cm	50(48.5)
T	
T0	0
T1	7(6.8)
T2	8(7.8)
T3	50(48.5)
T4	38(36.9)
N	
N0	58(56.3)
N1	24(23.3)
N2	21(20.4)
M	
M0	62(60.2)
M1	41(39.8)
Tumor classification	
I–II	73(70.9)
III–IV	30(29.1)
Vascular invasion	
No	83(80.6)
Yes	20(19.4)
Neurological invasion	
No	52(50.5)
Ye	51(49.5)
CEA	
≥ 5ng/ml	47(45.6)
< 5ng/ml	56(54.4)
CA199	
≥ 37U/ml	22(21.4)
< 37U/ml	81(78.6)

**Table 2:** Correlation between FTO expression and clinical charateristics of coloncancer patients(n = 103)

Clinical characteristics	FTO expression		*P*-value
	− ~ +	+ + ~ + +	
Age (years)			
≥ 65	8	44	0.28*
< 65	3	48	
Gender			
Male	3	54	0.048*
Femlae	8	38	
Tumor			
≥ 5cm	7	46	0.392
< 5cm	4	46	
T			
T0	0	0	0.017*
T1	0	7	
T2	3	5	
T3	2	48	
T4	6	32	
N			
N0	4	54	0.287
N1	3	21	
N2	4	17	
M			
M0	5	57	0.291
M1	6	35	
Tumor classification			
I–II	3	70	0.001**
III–IV	8	22	
Vascular invasion			
No	7	76	0.133
Yes	4	16	
Neurological invasion			
No	6	46	0.776
Yes	5	46	
CEA			
≥ 5ng/ml	5	42	0.894
< 5ng/ml	6	50	
CA199			
≥ 37U/ml	3	19	0.890
< 37U/ml	8	73	

*p < 0.05, **p < 0.01

### Overexpression of FTO enhances the processes of proliferation, migration, and invasion in colon cancer

**Fig. 3:**
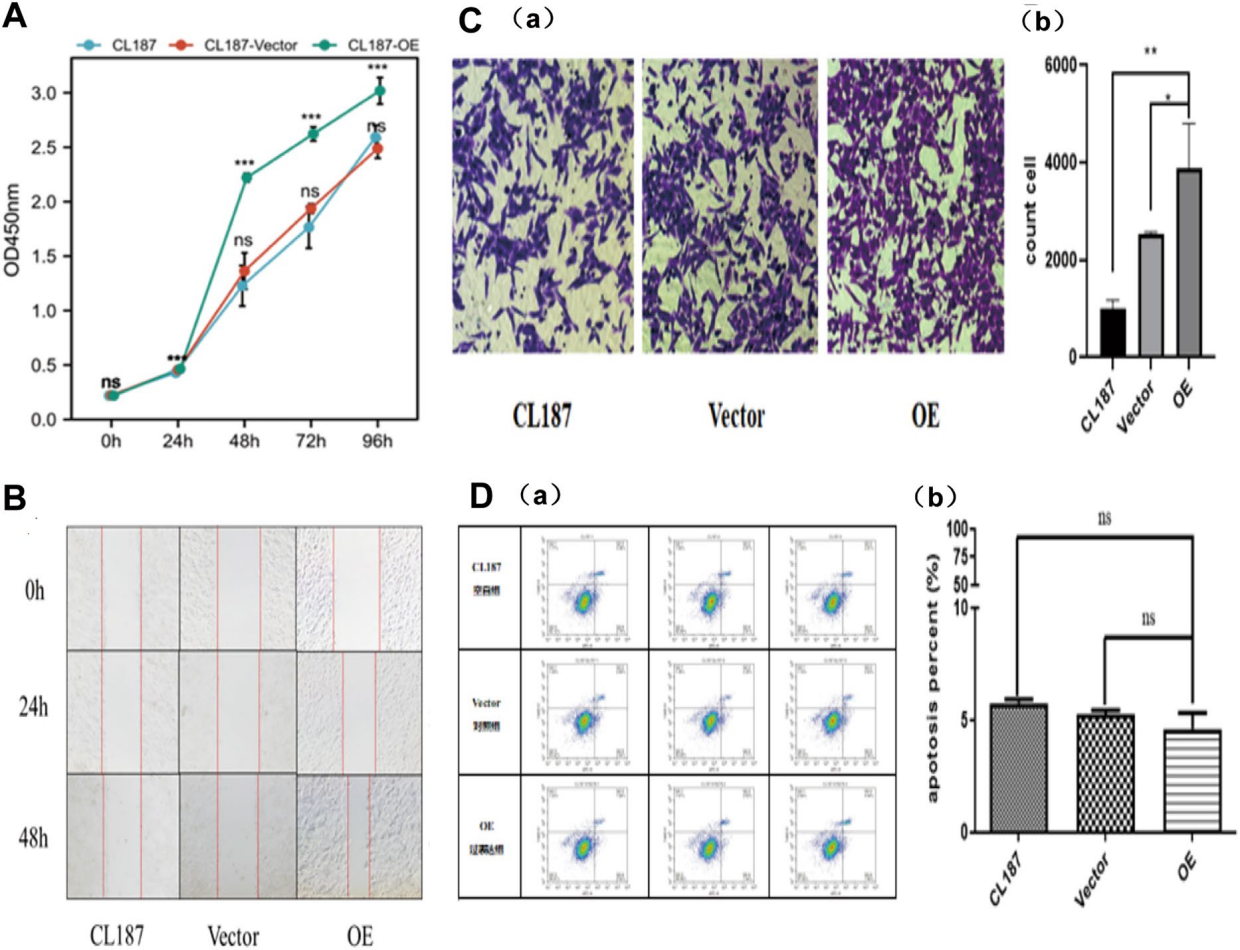
Overexpression of FTO promotes proliferation, migration, and invasion in colon cancer. **A**. In vitro, FTO overexpression stimulated the proliferation of colon cancer cells by the CCK-8 method. **B**. Compaed with the vector FTO, high expression of FTO significantly enhanced the proliferation. **C**. when FTO was highly expressed, aggressive migration and invasion of colon cancer cells were observed. **D**. In contrast, apoptosis in control vector cells was not significantly increased by the FTO overexpression.

Five colon cancer cell lines were employed to investigate FTO's biological function and examine the varying levels of FTO expression. Figure 1D demonstrates that LOVO exhibited high expression of FTO, while CL187 displayed low expression of FTO, both at the protein and mRNA levels. Following this, we investigated the effects of FTO overexpression on proliferation, migration, invasion, and apoptosis in the CL187 colon cell line. In vitro, the excessive expression of FTO in colon cancer cells stimulated proliferation as measured by the CCK-8 method (Fig. 3A). Notably, the high expression of FTO demonstrated a significant enhancement in proliferation compared to the vector FTO. Subsequently, the upregulation of FTO led to the observation of increased migration and invasion of colon cancer cells (Fig. 3B–C). Conversely, the overexpression of FTO did not lead to a notable rise in apoptosis in the control vector cells (Fig. 3D).

### Downregulation of FTO inhibits the processes of proliferation, migration, and invasion in colon cancer

**Fig. 4:**
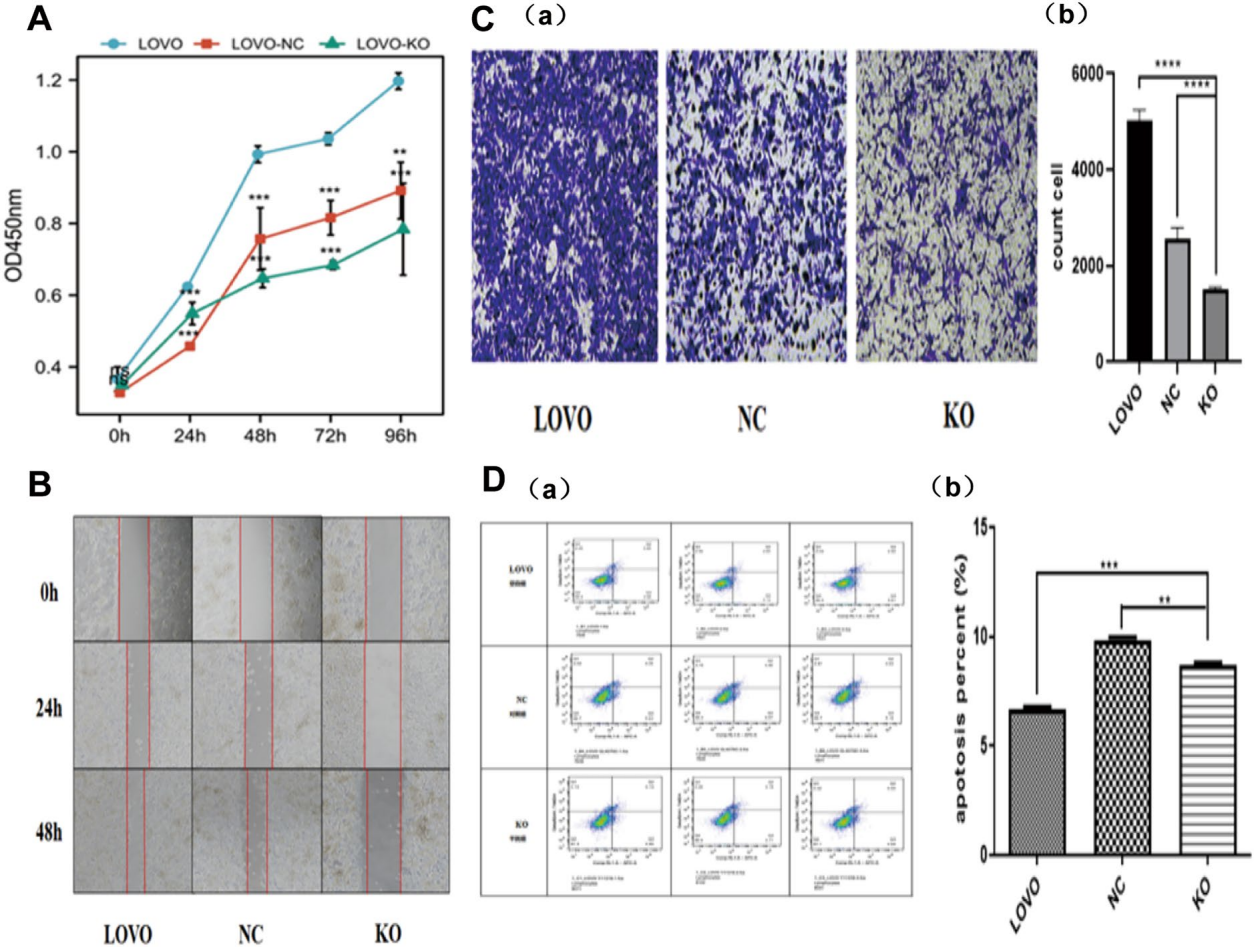
Downregulation of FTO inhibits proliferation, migration, and invasion in colon cancer.** A**. In vitro, FTO knockdown inhibited the proliferation of colon cancer cells corresponding to the empty vector. **B**. The downregulation of FTO expression suppressed cell migration. **C**. The transwell invasion assay confirmed that the knockdown of FTO inhibited cell invasion. **D**. The knockdown of FTO did not affect cell apoptosis. Although the difference between the knockdown FTO group and the empty vector group was statistically significant (P<0.01), apoptosis was in the normal range in LOVO cells knocked down by viewing the apoptosis images.

To acquire a more profound comprehension of the biological role of FTO, we examined the impacts of reducing FTO expression on cell proliferation, migration, invasion, and apoptosis in the LOVO cell line. It has been demonstrated that FTO knockdown inhibited the proliferation of colon cancer cells corresponding to the empty vector (Fig. 4A). Furthermore, the downregulation of FTO expression resulted in a notable suppression of cell migration (Fig. 4B). Subsequently, we examined the involvement of FTO in the modulation of cell invasion. Our study indicated that the transwell invasion assay provided evidence supporting the inhibitory impact of FTO knockdown on cell invasion (Fig. 4C). Conversely, the knockdown of FTO did not influence cell apoptosis. Although there was a statistically significant distinction between the FTO knockdown group and the empty vector group (P < 0.01), the levels of apoptosis in LOVO cells treated with FTO knockdown remained within the normal range, as confirmed by the analysis of apoptosis images (Fig. 4D).

### The impact of FTO on the expression of PKM2 in colon cancer cells

**Fig. 5:**
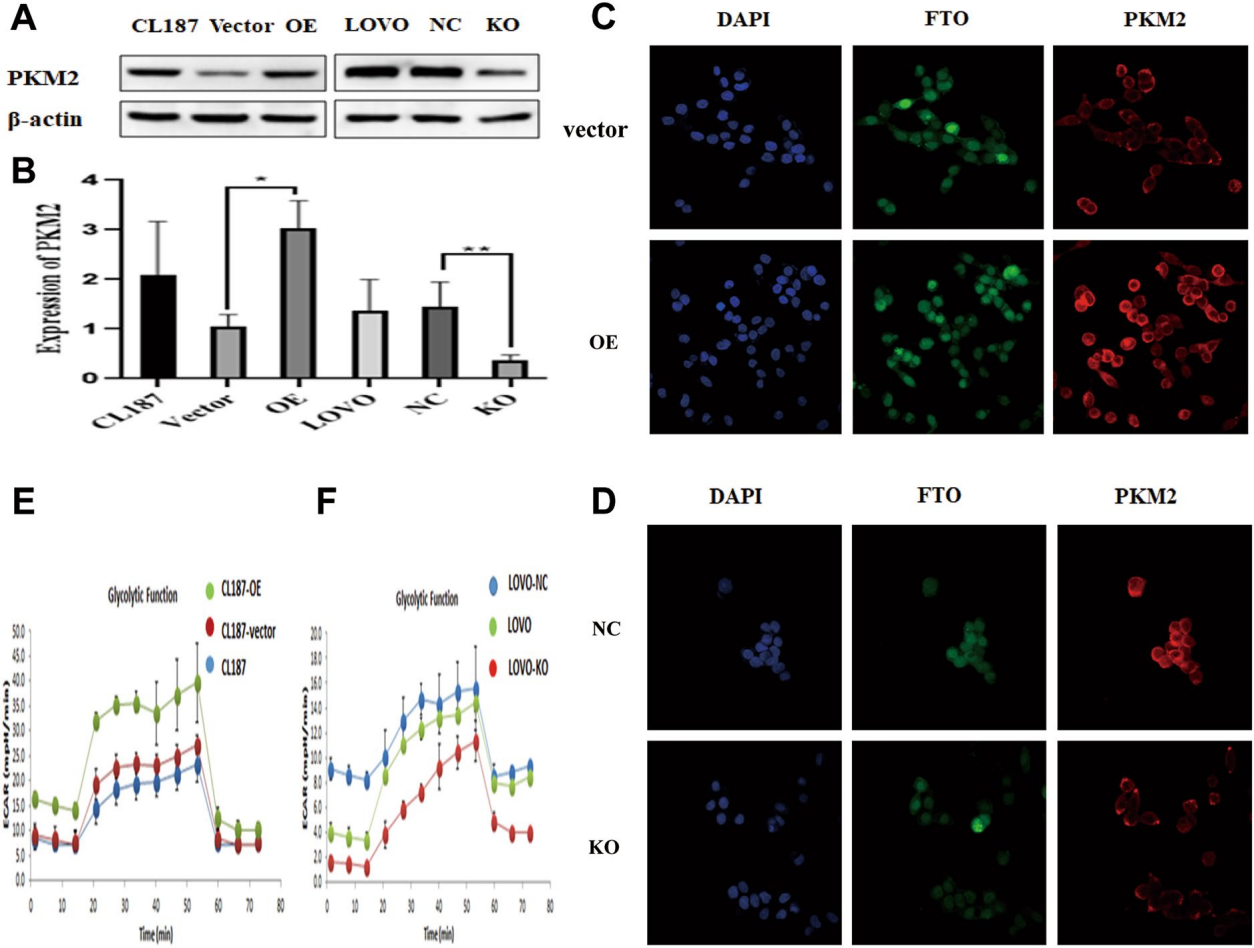
Effect of FTO on the expression of PKM2 in colon cancer cells. The expression of PKM2 was increased by overexpression FTO, Whereas the following knockdown FTO, the expression of PKM2 was suppressed compared with the empty vector from protein expression level **A** and RNA expression level **B**.When FTO was overexpressed in the CL187 cell line, the immunofluorescence staining of PKM2 was enhanced**C**. Conversely, the immunofluorescence staining of PKM2 was reduced with knockdown FTO in the LOVO cell line **D**. The FTO overexpression CL187 cells displayed significantly enhanced capacity for glycolysis in vivo, compared with the Vector control **E**. As compared to the control empty vector, the glycolysis capacity of colon cancer cells decreased when the LOVO cells were knocked down FTO (F).

In addition to investigating the influence of FTO overexpression or knockdown on biological function, our study aimed to explore additional metabolic traits that could be influenced by FTO expression. Given the crucial role of PKM2 in aerobic glycolysis, we subsequently manipulated FTO expression or silenced it to assess its effect on PKM2 expression. To ascertain the correlation between the two, we used western blot analysis to test the expression of PKM2 in colon cancer cells that overexpressed FTO or had FTO knocked down. As a result, there was a significant increase in PKM2 expression following FTO overexpression. In our study, the knockdown of FTO resulted in a suppression of PKM2 expression compared to the empty vector (Fig. 5A-B). In order to further explore the role of FTO in the aerobic glycolysis of colon cancer, we examined the expression of PKM2 in colon cancer cells using immunofluorescence staining about changes in FTO expression. The excessive expression of FTO in the CL187 cell line led to an enhancement in the immunofluorescence staining of PKM2.

In contrast, the knockdown of FTO in the LOVO cell line reduced the immunofluorescence staining of PKM2 (Fig. 5C-D). To elucidate the impact of energy metabolism on colon cancer, we investigated the expression of FTO using the Seahorse XF96 metabolic function analysis. Our findings indicated that the overexpression of FTO in CL187 cells led to a notable increase in glycolytic capacity in vivo, in contrast to the Vector control. Conversely, the glycolytic capacity of colon cancer cells decreased in LOVO cells when FTO was suppressed, compared to the control empty vector (Fig. 5E-F).

### PKM2 inhibitor suppresses the cell viability and glycolysis capacity of the colon cancer cells with overexpression FTO

Our study utilized a CCK-8 assay to investigate the impact of a PKM2 inhibitor on cell viability by modulating FTO expression. Initially, CL187 cells with overexpressed FTO were subjected to various concentrations (0.5 μM, 1.0 μM, 1.5 μM, 2.0 μM, 2.5 μM, 5 μM, and 7.5 μM) of the PKM2 inhibitor, shikonin, for a maximum duration of 24 and 48 h. Within 24 h, there was a concentration-dependent decrease in cell viability with increasing shikonin concentration, starting from 1.5 μM. Interestingly, a similar significant effect was observed after 48 h of treatment. The cell viability of CL187 cells with FTO overexpression exhibited a significant decline as the concentration of shikonin increased, particularly at concentrations of 1.0 μM and above. The findings from this study indicated that shikonin effectively inhibited the viability of colon cancer cells in a manner that depended on both time and dose. The IC50 value for cell viability after 24 h of treatment was determined to be 3.65 μM, while the IC50 value after 48 h was 1.84 μM (Supplementary Fig. 1A-C). Subsequently, shikonin concentrations of 1.5 μM and 2.5 μM were selected for further investigation of the glycolysis capacity of colon cancer cells. Our findings showed that the glycolysis capacity of colon cancer cells decreased when treated with shikonin at two specific concentrations for 24 h. The suppression of glycolysis capacity was dose-dependent, as higher concentrations of shikonin resulted in more significant suppression (Supplementary Fig. 1D). Additionally, the overexpression of FTO in these cells showed a significant decline in glycolysis capacity after being treated with 2.5 μM shikonin for 24 h, compared to the control vector.

## Discussion

Colon cancer is a common gastrointestinal tumor. Its complex evolution results from the synergistic effect of multiple genes and factors, and the evolution of the disease is complex. Although our understanding of colon cancer has deepened, its specific pathogenesis has not yet been fully elucidated. FTO, the first identified m6A demethylase and an allele associated with obesity, possesses demethylase activity. FTO can act on 3-methylthymine (3-meT) in single-stranded DNA and 3-methyluracil (3-meU) in single-stranded RNA (ssRNA)(Mayman et al. 2023). This demethylase activity may be bound up with the regulation of metabolism-related genes.

FTO plays diverse roles in different tumors, promoting or inhibiting tumorigenesis(Qiao et al. 2024). FTO not only plays a pivotal role in regulating metabolic rate, energy distribution, and lipid aggregation within the body, as well as facilitating stem cell differentiation(Pereira et al. 2021), but it is also intricately linked to the pathogenesis of cardiac and cerebrovascular diseases(Mathiyalagan et al. 2019), Alzheimer's disease(Han et al. 2020), schizophrenia(Morris et al. 2019; Pereira et al. 2021), and tumors (Yang et al. 2019; R et al. 2020). The research by Tao et al. demonstrated that FTO could promote the initiation and progression of bladder cancer through regulation of the MALA/miR-384/MAL2 axis via m6A modification, indicating that FTO has an active role in the tumorigenesis of bladder cancer. In the study of acute myeloid leukemia (AML), it was found that FTO can upregulate proteins such as PML-RARA and NPM1 to promote the occurrence and development of leukemia(Li et al. 2017).

Qing et al. (Qing et al. 2021) recently conducted a study that showed that the level of m6A in the transcripts of two pivotal metabolism-related target genes (PFKP and LDHB) was modulated by inhibiting FTO(Cui et al. 2021). This intervention led to an augmented degradation of mRNAs associated with these metabolism-related target genes, reducing their expression levels. Consequently, the inhibition of aerobic glycolysis was achieved in leukemia cells. While the precise mechanism by which FTO influences tumor development remains elusive, mounting evidence suggests a strong correlation between FTO and the incidence, progression, and prognosis of certain malignant neoplasms. Numerous studies have proven that FTO exhibits tumorigenic potential. Zhou et al. found that an excessive expression of FTO in individuals diagnosed with cervical cancer was linked to an unfavorable prognosis(Zhou et al. 2018). Furthermore, FTO plays a crucial part in the malignant characteristics of tumors. Another investigation into AML revealed that inhibiting FTO activity can impact the stability of MYC/CEBPA transcripts by up regulating the m6A expression. As a result, this suppresses the proliferation of AML cells(Su et al. 2018).

This also indicates FTO's significant impact on oncogenes(Li et al. 2017, 2020). Thus, we established a model of colon cancer cell lines stably transfected with high and low expression of FTO in our study. This enabled us to observe the influences of differential expression of FTO on cell proliferation, migration, invasion, and apoptosis. The results showed that overexpressing FTO enhanced the proliferation, migration, and invasion abilities of colon cancer cells, which consequently affected the malignant characteristics of these cells. This is similar to the findings of the studies above on the pathogenesis of acute myeloid leukemia, which confirmed FTO as an oncogenic factor in colon cancer. In recent years, the malignant biological behavior of FTO in tumors has been studied in multiple systems. For example, in digestive system tumors, it has been revealed that FTO exhibits significant expression in gastric cancer, and it promotes the initiation and progression of gastric cancer by augmenting the proliferation, migration, and lymphatic metastasis of gastric cancer cells. In reproductive system tumours, Zhao et al. (Zhao et al. 2020) found that FTO can enhance proliferation, inhibit apoptosis, and activate autophagy in ovarian cancer cells, thus affecting the occurrence and development of ovarian cancer. Moreover, the results obtained by Zhang et al. [24] showed that FTO could activate the WNT signalling pathway by catalyzing the demethylation of HOXB13 mRNA, thereby accelerating the metastasis and invasion of endometrial cancer. All of the results above are consistent with those found in our study. Specifically, FTO overexpression can motivate the migration and invasion of tumor cells, suggesting that FTO is a viral oncogene. To summarize, FTO plays a pro-cancer role in various tumors, and its high expression can affect the malignant biological behavior of tumor cells. With more in-depth studies of FTO in various tumors, it could serve as a predictive biomarker for tumors(Mi et al. 2023).

Research indicates that increased glycolysis drives tumor cell proliferation, facilitates metastasis, and contributes to resistance against radiotherapy and chemotherapy(Zhang et al. 2024). Glycolysis plays a critical role in the metabolic adaptation of CRC cells, particularly under hypoxic conditions, which are common in the tumor microenvironment. The intestinal environment, characterized by its substantial anaerobic milieu, further exacerbates hypoxia in CRC, promoting metabolic reprogramming that supports tumor growth and survival. Metabolic reprogramming is one of the common features of tumors(Dou et al. 2023), bringing advantages to tumor cells regarding growth, proliferation, migration, and metastasis by altering the efficiency and way nutrients such as glucose, amino acids, and fats are utilized. The most famous phenomenon of metabolic reprogramming in tumors is the "Warburg effect"—a phenomenon in which glucose metabolism is abnormally active in tumors. This phenomenon, or "aerobic glycolysis," is associated with activated oncogenes, such as RAS and CYC, and mutated tumor suppressor genes, including TP53. Its effects on tumors are primarily manifested in the significant proliferative capacity of tumor cells(Du et al. 2020; Wang et al. 2021). Under hypoxic conditions, tumor dependence on glycolysis can be further increased, and the hypoxic response can efficiently upregulate glucose transporters and multiple enzymes in the glycolytic pathway. Hypoxia-inducible factor 1-alpha (HIF-1α) is a critical transcription factor that becomes stabilized under hypoxic conditions. It plays a pivotal role in the adaptive response to low oxygen levels by upregulating the expression of key glycolytic enzymes, such as hexokinase 2 and pyruvate kinase M2, as well as glucose transporters like GLUT1. This upregulation enhances the glycolytic capacity of colorectal cancer CRC cells, allowing them to maintain energy production even in the absence of sufficient oxygen(Thamrongwaranggoon et al. 2023; Chen et al. 2024). The increased glycolytic flux under hypoxia leads to excessive lactate production, which acidifies the tumor microenvironment. This acidic environment promotes angiogenesis, immune suppression, and extracellular matrix remodeling, facilitating TAM Ap-2alpha activity and CRC progression(Wang et al. 2020, 2024). Hypoxia and glycolysis are closely linked to the maintenance of cancer stem cells (CSCs) in CRC. CSCs rely on glycolysis for energy production under hypoxic conditions, which enhances their self-renewal capacity and resistance to chemotherapy(Zhong et al. 2024).

Many isozymes exist in the sugar metabolic pathway. Analyzing the differential expression of these isozymes in tumor and normal cells is one of the important approaches in tumor glucose metabolism research. Of these isozymes, the study of differential expression of pyruvate kinase (Pyruvate Kinase M, PKM) has attracted the most attention. Pyruvate kinase plays a crucial role in the glycolytic pathway by catalyzing the production of pyruvate and ATP metabolites. The gene encoding PKM can express two isozymes, PKM1 and PKM2, by selecting either the ninth or tenth exon using differential mRNA splicing. The expression of PKM1 and PKM2 varies significantly: PKM1 is mainly expressed in terminally differentiated cells with relatively high energy demands, such as muscle and brain, while PKM2 is mainly expressed in cells with relatively strong anabolic demands, such as embryonic cells, stem cells, and tumor cells. Although both catalyze the same reaction, the difference of only 22 amino acids confers different properties to PKM1 and PKM2, including multimerization, enzyme activity, and mode of regulation.

The tumor microenvironment is recognized as an essential factor and condition for sustaining tumor cell growth(Guo et al. 2023). Moreover, it is closely associated with tumor energy metabolism. Glucose metabolism is the primary pathway by which cells obtain energy and maintain growth for cells; however, the conversion from normal cells to tumor cells is often accompanied by the remodeling of metabolic pathways. Typically, proliferating tumor cells break down glucose at an unexpectedly high rate, converting most of the glucose to lactate rather than oxidizing it to carbon dioxide, even in excess oxygen. The increase in glycolysis can transfer its intermediates to the synthetic pathways of various substances, including nucleotide and amino acid production pathways, which are required for active cell proliferation. A growing body of research has recently been dedicated to developing advanced anticancer therapies that leverage the differences in metabolic phenotypes between cancer and normal cells(Qing et al. 2021). Moreover, it has been proven that vital metabolic enzymes in different metabolic pathways can be safely targeted. In this study, we found that FTO affects the glycolysis level of colon cancer cells primarily by adjusting the expression level of PKM2, which provides novel insights for colon cancer therapy. In the glycolytic pathway, PKM2 acts downstream of PFKP, and the effect of FTO on PKM2 may be mediated through PFKP. In a study where R-2HG was shown to attenuate aerobic glycolysis in leukemia by targeting the FTO/m6A/PFKP/LDHB axis(Qing et al. 2021), it was found that PFKP promotes glycolysis and exerts an oncogenic role in leukemia. Research on its mechanism revealed that owing to the suppression of FTO by R-2HG, the m6A levels on the transcripts of two key enzymes of glycolysis, PFKP and LDHB, were upregulated. This, consequently, increased the degradation of the mRNAs of these two metabolic target genes and decreased their expression, thus selectively inhibiting aerobic glycolysis. Our study showed that differential expression of FTO could cause differential expression of PKM2, and the two showed a positive correlation. Notably, after overexpression of FTO, the protein level of PKM2 in the colon cancer cell group increased than that of the empty group. When FTO was under-expressed, the protein level of PKM2 was decreased. As PKM2 serves as a critical molecule downstream of the vital enzyme PFKP in the glycolytic pathway, it is hypothesized that the regulatory effect of FTO on PKM2 in colon cancer is likely to be exerted through the modification of PFKP at the m6A level by FTO, thereby indirectly affecting the expression of PKM2. Recent studies have demonstrated that PFKP mRNA is subject to m6A modification, which affects its stability and translation(Fang et al. 2022). Conversely, the Seahorse assay was applied to detect the glycolysis level of cells with differential FTO expression. The results also indicated that FTO overexpression could enhance the glycolysis level of colon cancer cells, and when underexpressed, it could reduce the glycolysis level of colon cancer cells. In summary, this study revealed that FTO can affect aerobic glycolysis in colon cancer by influencing the expression level of PKM2, confirming the linkage between FTO and PKM2. Additionally, the study by Li et al.(Li et al. 2019) Regarding hepatocellular carcinoma, it was shown that FTO could promote the translation of PKM2 by triggering the demethylation of PKM2 at the mRNA level, which also confirmed the correlation between the two genes. The specific mechanism of the linkage requires further investigation in the future. The administration of Shikonin, an inhibitor of PKM2, could suppress the glycolysis level in colon cancer cells, inhibit their proliferation, and influence the malignant characteristics of colon cancer at the level of energy metabolism. We recognize that there are limitations to our research. First, the absence of animal model experiments constrains our capacity to directly evaluate the functional impact of FTO on tumorigenesis and metastasis in a living organism. It is imperative that future research endeavours include animal experiments to bolster our current findings and to achieve a more holistic appreciation of FTO's biological significance in the context of CRC. Second, despite having access to local center data, we were unable to perform survival analysis. This limitation hinders our capacity to fully assess the influence of FTO on postoperative survival outcomes in CRC patients. Subsequent studies should focus on enhancing the use of follow-up data within local patient cohorts to more accurately determine the prognostic significance of FTO in CRC. Therefore, FTO, as a molecule significantly associated with metabolism, can affect the level of energy metabolism, especially glycolysis, in colon cancer. The findings of this study also provide a new theoretical basis and research direction for exploring the treatment of colon cancer.

## Conclusion

The impact of FTO on colon cancer glycolysis has significant implications for colon proliferation, invasion, and metastasis, as it positively modulates the expression of PKM2. It offers a novel therapeutic approach for targeting colon cancer metabolism.

## Supplementary Information

Below is the link to the electronic supplementary material.

Supplementary Figure 1. PKM2 inhibitor suppresses the cell viability and glycolysis capacity of the colon cancer cells with overexpression FTO. A. The cell viability of CL187 cells with FTO overexpression showed the most apparent decrease with increasing shikonin concentrations, especially 1.0μM above. B. The IC50 value of cells within 24 hours of treatment was 3.65μM. C. The IC50 value of cells within 48 hours of treatment was 1.84μM. D. The capacity of glycolysis was suppressed as shikonin concentrations increased . Meanwhile, the glycolysis capacity of overexpression FTO showed significant declines within 24 hours of 2.5μM shikonin treatment, compared to the vector. Supplementary file1 (TIF 1075 KB)

Supplementary file2 (TIF 1074 KB)

## Data Availability

No datasets were generated or analysed during the current study.

## Abbreviations

*FTO*: Fat mass and obesity-associated protein
*PKM2*: Pyruvate kinase isotype M2
*CRC*: Colorectal cancer
*TCGA*: The cancer genome atlas
*AML*: Acute myeloid leukemia
*PKM*: Pyruvate kinase M
*m6A*: N6-methyladenosine
