# Supplementary figures and images for: FTO effects the proliferation, invasion, and glycolytic metabolism of colon cancer by regulating PKM2
Source: J Cancer Res Clin Oncol. 2025 Jan 16;151(1):36. doi: 10.1007/s00432-024-06073-x (PMC11739181; doi:10.1007/s00432-024-06073-x)

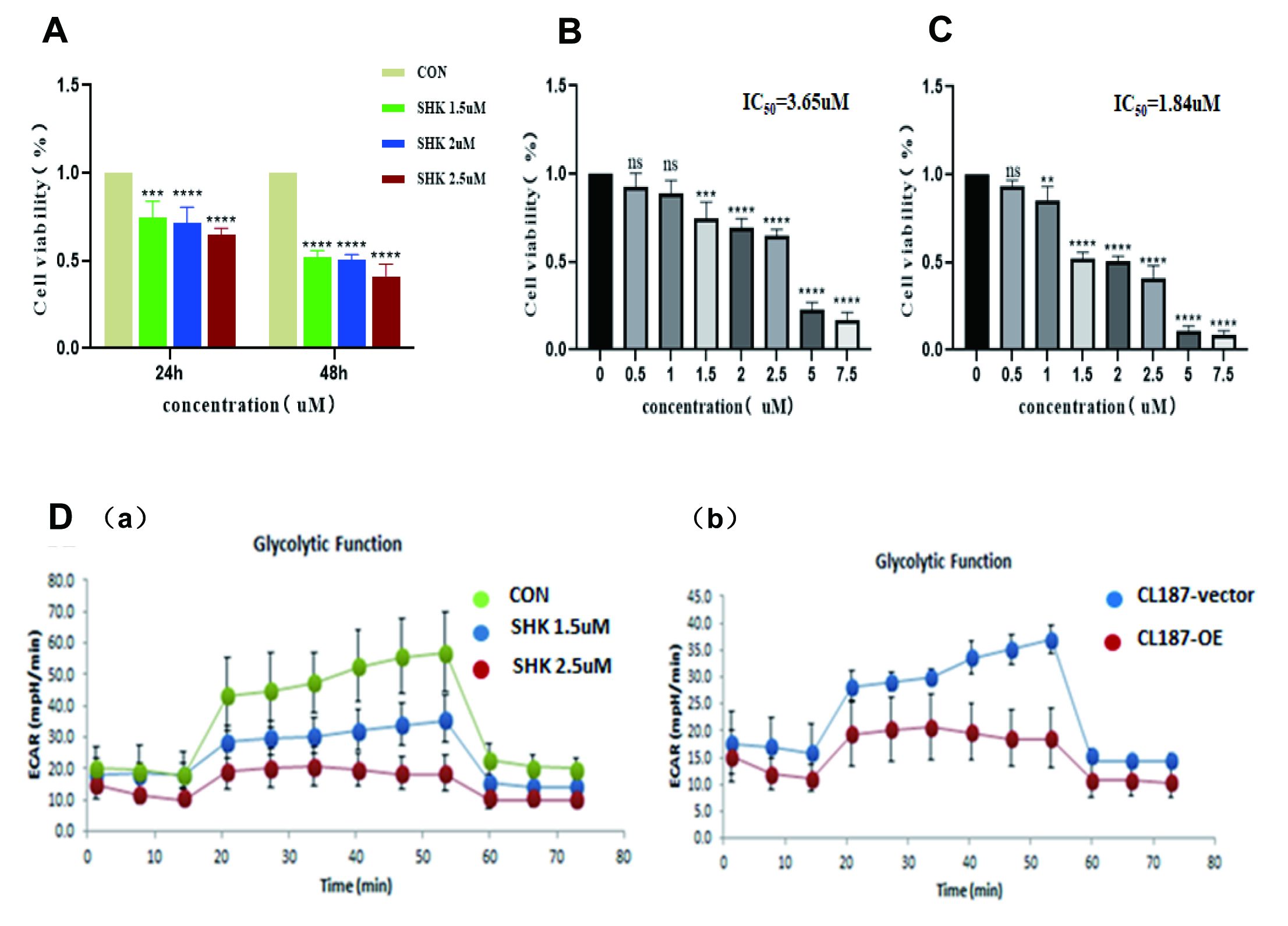

Supplement: Supplementary file 1 — Supplementary Figure 1. PKM2 inhibitor suppresses the cell viability and glycolysis capacity of the colon cancer cells with overexpression FTO. A. The cell viability of CL187 cells with FTO overexpression showed the most apparent decrease with increasing shikonin concentrations, especially 1.0μM above. B. The IC50 value of cells within 24 hours of treatment was 3.65μM. C. The IC50 value of cells within 48 hours of treatment was 1.84μM. D. The capacity of glycolysis was suppressed as shikonin concentrations increased . Meanwhile, the glycolysis capacity of overexpression FTO showed significant declines within 24 hours of 2.5μM shikonin treatment, compared to the vector. Supplementary file1 (TIF 1075 KB) [file 432_2024_6073_MOESM1_ESM.tif]

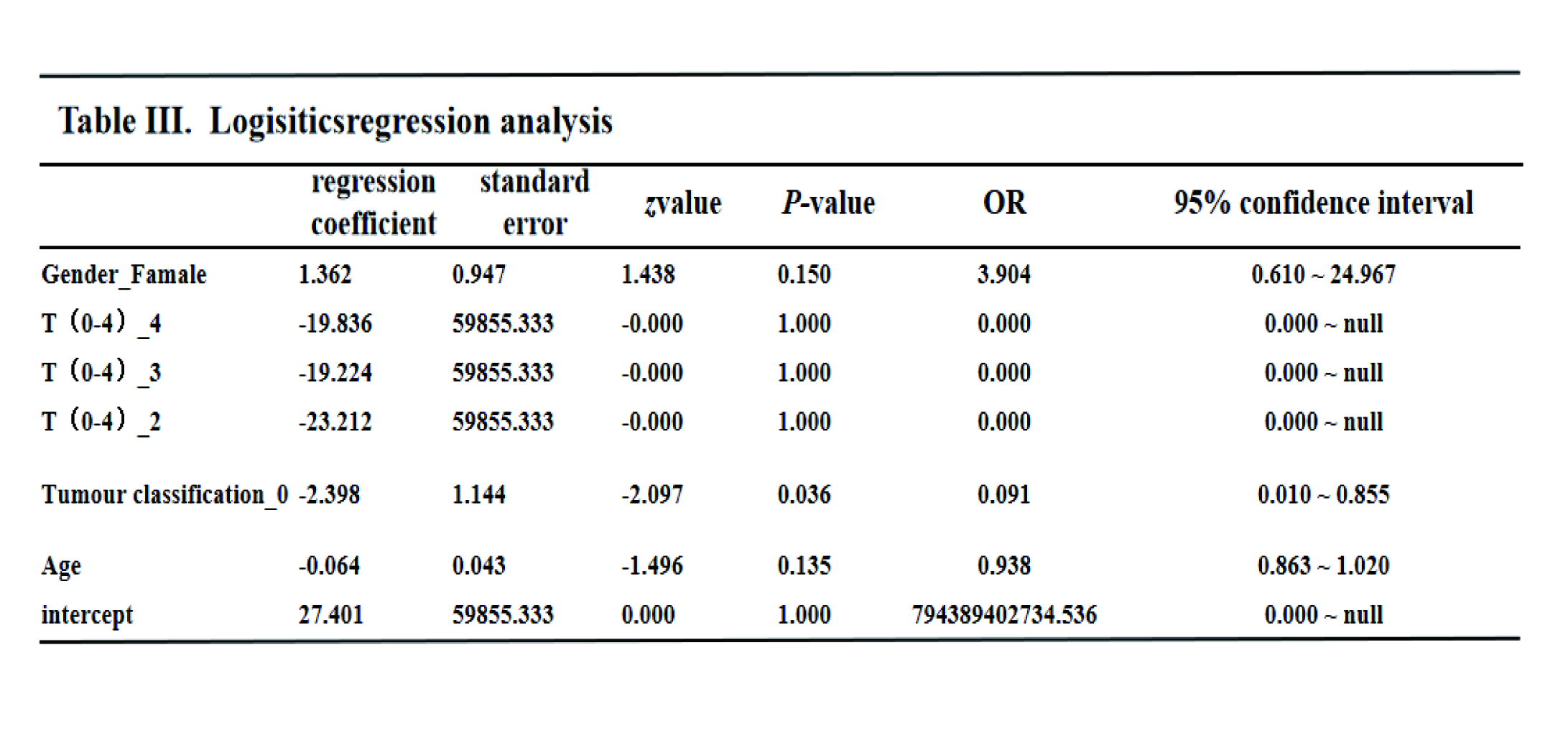

Supplement: Supplementary file 2 — Supplementary file2 (TIF 1074 KB) [file 432_2024_6073_MOESM2_ESM.tif]
